# Glutamate Dehydrogenase Is Important for Ammonia Fixation and Amino Acid Homeostasis in Brain During Hyperammonemia

**DOI:** 10.3389/fnins.2021.646291

**Published:** 2021-06-16

**Authors:** Caroline M. Voss, Lene Arildsen, Jakob D. Nissen, Helle S. Waagepetersen, Arne Schousboe, Pierre Maechler, Peter Ott, Hendrik Vilstrup, Anne B. Walls

**Affiliations:** ^1^Department of Drug Design and Pharmacology, Faculty of Health and Medical Sciences, University of Copenhagen, Copenhagen, Denmark; ^2^Department of Cell Physiology and Metabolism, Faculty of Medicine, University of Geneva, Medical Centre, Geneva, Switzerland; ^3^Department of Hepatology and Gastroenterology, Aarhus University Hospital, Aarhus, Denmark

**Keywords:** glutamate dehydogenase, brain, hyperammonemia, glutamate, alanine, pyruvate carboxylase (PC), glutamine

## Abstract

Impaired liver function may lead to hyperammonemia and risk for hepatic encephalopathy. In brain, detoxification of ammonia is mediated mainly by glutamine synthetase (GS) in astrocytes. This requires a continuous *de novo* synthesis of glutamate, likely involving the action of both pyruvate carboxylase (PC) and glutamate dehydrogenase (GDH). An increased PC activity upon ammonia exposure and the importance of PC activity for glutamine synthesis has previously been demonstrated while the importance of GDH for generation of glutamate as precursor for glutamine synthesis has received little attention. We therefore investigated the functional importance of GDH for brain metabolism during hyperammonemia. To this end, brain slices were acutely isolated from transgenic CNS-specific GDH null or litter mate control mice and incubated in aCSF containing [U-^13^C]glucose in the absence or presence of 1 or 5 mM ammonia. In another set of experiments, brain slices were incubated in aCSF containing 1 or 5 mM ^15^N-labeled NH_4_Cl and 5 mM unlabeled glucose. Tissue extracts were analyzed for isotopic labeling in metabolites and for total amounts of amino acids. As a novel finding, we reveal a central importance of GDH function for cerebral ammonia fixation and as a prerequisite for *de novo* synthesis of glutamate and glutamine during hyperammonemia. Moreover, we demonstrated an important role of the concerted action of GDH and alanine aminotransferase in hyperammonemia; the products alanine and α-ketoglutarate serve as an ammonia sink and as a substrate for ammonia fixation *via* GDH, respectively. The role of this mechanism in human hyperammonemic states remains to be studied.

## Introduction

Ammonia is a product of protein and amino acid metabolism and in the healthy liver ammonia is irreversibly removed by formation of urea which is subsequently excreted in the urine. Decreased liver function leads to impaired urea synthesis capacity (Vilstrup, 1984) and when porto-systemic shunts are present, ammonia-rich blood from the intestines bypasses the liver. These disturbances result in systemic and cerebral hyperammonemia, associated with a risk for hepatic encephalopathy, which is the most deleterious complication to liver diseases. The amount of ammonia entering the brain is determined by the arterial blood ammonia levels (Sorensen and Keiding, 2007). Ammonia is toxic to the brain through mechanisms involving secondarily disturbed metabolism and eventually neurotransmission, manifesting as hepatic encephalopathy. This is a neuropsychiatric syndrome, which occurs as a complication to acute and chronic liver failure, with symptoms ranging from mild cognitive impairment to coma (Vilstrup et al., 2014). With very high ammonia concentrations in acute liver failure there is a high risk for cerebral herniation (Ott et al., 2005).

*In vivo* as well as *in vitro* studies demonstrate that the brain disposes of ammonia mainly by astrocytic synthesis of glutamine that is exported from the brain, and to a lesser extent by glutamate synthesis (Norenberg and Martinez-Hernandez, 1979; Sibson et al., 2001; Zwingmann et al., 2003; Dadsetan et al., 2013; Thrane et al., 2013). Since ammonia fixation into the amide nitrogen of glutamine, catalyzed by glutamine synthetase (GS), is an adenosine triphosphate (ATP) dependent reaction, hyperammonemia imposes an energy demand on astrocytes with consequences for cellular energy status and energy metabolism. When GS is inhibited by methionine sulfoximine (MSO), there is a compensatory increase in alanine production, catalyzed by the concerted action of glutamate dehydrogenase (GDH) and alanine aminotransferase (ALAT) (Dadsetan et al., 2013). In order to sustain *de novo* synthesis of glutamate, the activity of both the astrocyte specific enzyme pyruvate carboxylase (PC) (Yu et al., 1983; Shank et al., 1985; Schousboe et al., 2019) and GDH is required. The importance of PC for glutamine synthesis and the consequent ammonia fixation has been quite extensively studied *in vitro* and *in vivo* (Berl et al., 1962; Lapidot and Gopher, 1997; Zwingmann et al., 1998, 2003; Kanamatsu and Tsukada, 1999; Leke et al., 2011). In contrast, the importance of GDH for generation of glutamate to be used as substrate for glutamine synthesis has received little attention.

Glutamate dehydrogenase (GDH) is a mitochondrial enzyme that catalyzes either the oxidative deamination of glutamate to α-ketoglutarate or, conversely, the reductive amination of α-ketoglutarate, forming glutamate. While several aminotransferases catalyze the reversible interconversion between glutamate and α-ketoglutarate, GDH is unique by catalyzing a reaction that leads to a net supply of TCA cycle intermediates, i.e., anaplerosis, and at the same time playing an essential role in ammonia homeostasis by releasing or fixating ammonia from or into glutamate α - amino nitrogen. Glutamate dehydrogenase is subject to complex allosteric regulation including activation by ADP (Li et al., 2012) thereby facilitating glutamate oxidation upon a reduction in the cellular energy status. In line with this, cultured astrocytes lacking GDH revealed significant changes in cellular energetics (Karaca et al., 2015) and glutamate metabolism (Frigerio et al., 2012). Rodents express only one isoform of GDH which is encoded by the gene *Glud1* (Michaelidis et al., 1993) and in the brain GDH is expressed in both neurons and astrocytes (Rothe et al., 1994; McKenna et al., 2000). However, only astrocytes and not neurons express GS (Norenberg and Martinez-Hernandez, 1979). Thus, neuronal ammonia metabolism relies exclusively on GDH-catalyzed amination of α-ketoglutarate to form glutamate. Although sparsely investigated, this may mean that GDH activity is likely to be crucial for neuronal survival during hyperammonemia.

The present study was conducted to investigate the functional role of GDH with regard to ammonia fixation and amino acid homeostasis in brain during hyperammonemia. To this end we used a transgenic CNS-specific GDH null (*Glud1^–/–^*) and litter mate control *Glud1*^*lox/lox*^ mice. Acutely isolated brain slices from these mice were incubated in medium containing [U-^13^C]glucose in the absence or presence of ammonia or containing ^15^N-labeled ammonia in the presence of glucose. Our results reveal a central importance of GDH function for cerebral ammonia fixation and as a prerequisite for synthesis of glutamine during hyperammonemia.

## Materials and Methods

### Materials

Sodium tetraborate decahydrate, bicinchoninic acid disodium salt hydrate, and *N,N*-Dimethylformamide (DMF), *N-tert*-butyldimethylsilyl-*N*-methyltrifluoroacetamide (MTBSTFA) were purchased from Sigma Aldrich (St. Louis, United States). Sodium azide was purchased from Merck KGaA (Darmstadt; Germany). Micro BCA^TM^ Reagent A (MA) and Micro BCA^TM^ Reagent C (MC) were purchased from Thermo Scientific (Rockford, United States). Methanol (≥ 99.8%, filtered at 0.2 μm) and acetonitrile (≥99.9% filtered at 0.2 μm) gradient grades for high performance liquid chromatography (HPLC) were purchased from VWR International is the name of the company (Fortenay-sous-Bois, France). Ampoules with *o*-phthaldehyde (OPA) and 3-mercaptopropionic acid in 0.4 M borate buffer and borate buffer was purchased from Agilent Technologies (Santa Clara, United States) and 15NH4Cl (99% [15N] enriched) was purchased from Cambridge Isotope Laboratories (Tewksbury, United States). D-[U-^13^C]glucose (99% [^13^C] enriched) was produced by Isotec (Miamisburg, United States). All other chemicals used were of the purest grade available from regular commercial sources.

### Mice

The CNS-specific GDH null mice (CNS-*Glud1^–/–^*) were initially obtained from Professor Pierre Maechler’s laboratory, The Medical Faculty, University of Geneva, Switzerland and subsequently housed and bred in the animal facility at Department of Drug Design and Pharmacology, University of Copenhagen (Copenhagen, Denmark). In brief, CNS specific CNS-*Glud1^–/–^* mice were generated by crossing floxed *Glud1* mice (*Glud1*^*lox/lox*^) with mice expressing Nestin-*Cre* transgene where *Cre* recombinase was under control of a Nestin regulatory sequence (Tronche et al., 1999; Carobbio et al., 2009). Subsequently, Nestin-Cre mice were crossed with Glud1*^*lox/lox*^* mice to generate *Glud1^*lox/lox*^ x Cre*^±^ (CNS-*Glud1^–/–^*) mice. An inbred strain-specific phenotype was avoided by maintaining the mice on a mixed genetic background (C57BL/6J x 129/Sv). Heterozygous breeding led to equal generation of *Glud1^*lox/lox*^ x Cre*^±^ (CNS-*Glud1^–/–^*) and littermate *Glud1^*lox/lox*^ x Cre^–/–^* (*Glud1*^*lox/lox*^) which were used as control animals. Acutely isolated brain slices were prepared from mice aged 9–20 weeks and both genders were used. The use of control and knockout mice with a mixed homogenous genetic background at the age of 9–20 weeks avoids potential effects on body weight contributed by the Nestin-Cre transgene (Karaca and Maechler, 2014). Also, a previous characterization of the CNS-*Glud1^–/–^* mice, revealed no gender differences (Frigerio et al., 2012; Karaca and Maechler, 2014).

### Slice Preparation and Incubation

Following cervical dislocation, the mice were decapitated, and isolated brains were immediately submerged into ice-cold artificial cerebrospinal fluid (aCSF, 5 mM D-glucose, 25 mM NaHCO_3_, 128 mM NaCl, 2 mM CaCl_2_ ⋅ 2 H_2_O, 3 mM KCl, 1.2 mM MgSO_4_ and 0.4 mM KH_2_PO_4_, pH 7.4 ± 0.1; Tranberg et al., 2004). The hippocampi and cortices were dissected out while kept in ice-cold aCSF and the brain regions were sliced into slices of 350 μm using a McIllwain tissue chopper (The Vibratome Company, O’Fallon, MO, United States). The slices were separated under microscope while kept in ice-cold aCSF and subsequently the slices were transferred to baskets in a specially designed incubation system (McNair et al., 2017). Four to six hippocampal slices or two cortical slices were used per condition. The slices were kept at the surface of 10 mL oxygenated aCSF and pre-incubated for 60 min at 37°C. After 60 min of pre-incubation the medium was changed to aCSF where unlabeled glucose was substituted by 5 mM [U-^13^C]glucose. Cortex and hippocampus slices were incubated in the absence or presence of ammonia (1 or 5 mM) for 60 min at 37°C. In another set of experiments employing the same experimental conditions, cortex slices were incubated in aCSF containing 1 or 5 mM ^15^N-labeled NH_4_Cl in the presence of 5 mM unlabeled glucose.

### GC-MS Analysis

The percentage of ^13^C labeling in alanine, citrate, glutamate, malate, and aspartate was determined using a method from Mawhinney et al. (1986) and described in detail in Walls et al. (2014). Briefly, following reconstitution in water, an aliquot of the lyophilized slice extracts was adjusted to pH 1–2 and evaporated to complete dryness under a nitrogen flow. Organic extraction was performed using 96% ethanol and benzene followed by evaporation to complete dryness, and this procedure was repeated. Organic and amino acids in the extracts were derivatized using *N-tert*-butyldimethylsilyl-*N*-methyl-trifluoroacetamide in the presence of dimethylformamide. The samples were analyzed by gas chromatography (GC; Agilent Technologies 7820A, J&W GC column HP-5MS) coupled to a mass spectrometer (MS; Agilent Technologies 5977E). Isotopic labeling was corrected for natural abundance determined from an unlabeled standard solution containing the metabolites of interests, and the percentage of M + X, where M is the parent ion and X is the number of ^13^C labeled atoms, was calculated as described by Biemann (Biemann, 1962) and Walls et al. (Walls et al., 2014).

### HPLC Analysis

The content of the amino acids alanine, glutamate, aspartate and γ-aminobutyric acid (GABA) in slice extracts was measured by reversed phase high performance liquid chromatography (HPLC; Agilent Technologies 1260 Infinity). The lyophilized extracts were dissolved in water and an aliquot was derivatized with *o*-phthaldehyde and detected using fluorescent detection (excitation λ: 338 nm, emission λ: 390 nm). The amino acids were separated on a Zorbax Eclipse Plus C18 column (4.6 × 150 mm, 3.5 μm) and a mobile phase gradient of mobile the phases A (10 mM Na_2_HPO_4_, 10 mM Na_2_B_4_O_7_ ⋅ 10 H_2_O and 0.5 mM NaN_3_, pH 6.8) and B [Acetonitrile 45%: Methanol 45%: H_2_O 10% (v:v:v)]. The percentage of mobile phase B increased linearly from 2 to 57% in 30 min, from 57 to 100% in 0.1 min and then returned to 2% in the last period. The total run time was 35 min, and the flow was maintained at 1.5 ml/min throughout the run. The amounts of amino acids in the samples were calculated from standards containing the amino acids of interest and related to protein content in the samples.

### ^15^N Labeling From ^15^NH_4_Cl

When employing ^15^N-labeled NH_4_Cl, the labeled ammonia will be traceable, thereby revealing the biochemical pathways involved in cerebral ammonia detoxification. ^15^NH_4_ can be fixated into glutamate or glutamine by the enzymes GDH and GS, respectively, leading to mono-labeled (M + 1) glutamate and glutamine. Following fixation into glutamate, the ammonia group may be transferred to aspartate and alanine in the transamination reactions catalyzed by aspartate aminotransferase (AAT) and ALAT, respectively, resulting in mono-labeling (M + 1) in aspartate and alanine. In addition, glutamate is precursor for GABA and ^15^N labeling in GABA may occur following fixation into glutamate. Hence, mono-labeling (M + 1) in glutamate, aspartate, alanine and GABA occurs following fixation of ^15^NH_4_ into glutamate. As glutamate is also the precursor of glutamine, glutamine may be mono-labeled as a result of ^15^NH_4_ fixation either in the GDH or the GS reaction while double-labeling occurs if ^15^NH_4_ is fixated in both reactions. In the GDH deficient mice, ammonia fixation is dependent upon GS alone and it is therefore not possible to obtain labeling in glutamate, aspartate, alanine or GABA, and mono-labeling will only be present in glutamine.

### ^13^C Labeling From [U-^13^C]glucose

[U-^13^C]Glucose is *via* glycolysis converted to [U-^13^C]pyruvate (M + 3) which may be converted to lactate M + 3 or aminated to alanine M + 3 by ALAT; the latter process concomitantly converts glutamate to α-ketoglutarate. In astrocytes, pyruvate M + 3 may be carboxylated to generate oxaloacetate M + 3, a process catalyzed by the astrocyte specific enzyme PC (Schousboe et al., 2019). This process is important for *de novo* synthesis of amino acids, and hence is involved in the maintenance of (neurotransmitter) amino acid homeostasis. Alternatively, pyruvate M + 3 may enter the TCA cycle either *via* pyruvate dehydrogenase (PDH) to form acetyl coenzyme A M + 2 which following condensation with oxaloacetate generates citrate M + 2. It should be noted that the concerted actions of PC and PDH gives rise to citrate M + 5. Citrate M + 2 is in the TCA cycle converted to α-ketoglutarate M + 2 which, by means of an aminotransferase or GDH, may be converted to glutamate M + 2. In astrocytes, glutamate M + 2 is precursor for glutamine M + 2, a process catalyzed by the astrocyte specific enzyme GS (Norenberg and Martinez-Hernandez, 1979) while in GABAergic neurons glutamate M + 2 may be converted to GABA M + 2. Alternatively, α-ketoglutarate M + 2 may be further metabolized in the TCA cycle to form succinate M + 2, fumarate M + 2, malate M + 2, and oxaloacetate M + 2. Transamination of oxaloacetate M + 2 leads to aspartate M + 2, a process concomitantly converting glutamate to α-ketoglutarate.

### Data Analysis

Data are presented as averages ± standard error of the mean (SEM). Statistically significant differences between CNS-*Glud1^–/–^* and *Glud1*^*lox/lox*^ control mice were determined using an unpaired Student’s *t*-test while effects of ammonia treatment within each genotype was determined employing a one-way analysis of variance (ANOVA) followed by a Tukey’s multiple comparisons *post hoc* test. The significance level was set at *p* < 0.05.

## Results

### Metabolic Changes in Brain Slices From CNS-*Glud1^–/–^* Mice

The content of amino acids was determined in cortex and hippocampus slices from CNS-*Glud1^–/–^* and *Glud1*^*lox/lox*^ mice incubated in the absence of ammonia. The amount of alanine was significantly increased in hippocampal slices from CNS-*Glud1^–/–^* mice while the contents of glutamate, glutamine, aspartate, and GABA were similar in the two genotypes (Table 1). The increase in alanine could suggest a higher ALAT activity in order to compensate for the lack of GDH.

**TABLE 1:** The amounts (nmol/mg protein) of the metabolites alanine, aspartate, glutamate, glutamine, and GABA in extracts from hippocampal and cortex slices from *Glud1*^*lox/lox*^ and CNS-*Glud1^–/–^* mice was determined by high performance liquid chromatography (HPLC).

Amounts (nmol/mg protein) of amino acids in hippocampal slice extracts						

Brain area	Genotype	Alanine	Aspartate	Glutamate	Glutamine	GABA
Hippocampus	*Glud1*^*lox/lox*^	1.68 ± 0.34	72.5 ± 8.4	260.6 ± 23.2	9.90 ± 1.45	21.6 ± 2.4
	*Glud1^–/–^*	4.69 ± 0.66*	69.3 ± 8.8	290.6 ± 29.7	9.00 ± 1.09	21.2 ± 2.3
Cortex	*Glud1*^*lox/lox*^	5.10 ± 0.92	73.0 ± 9.2	268.3 ± 30.9	13.4 ± 1.97	21.6 ± 2.7
	*Glud1^–/–^*	3.62 ± 0.71	72.0 ± 14.8	298.1 ± 36.5	8.58 ± 1.12	19.0 ± 2.5

*The results are presented as averages ± SEM based on measurement of slices from 9 to 10 individual mice. Statistically significant differences (*P* < 0.05) between *Glud1*^*lox/lox*^ and CNS-*Glud1^–/–^* mice are indicated with an asterisk.*

**FIGURE 1:**
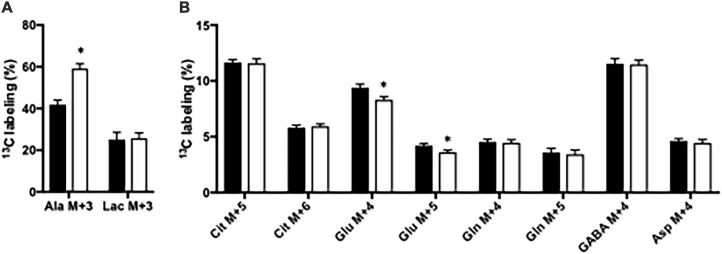
The percent ^13^C labeling in metabolites in hippocampal slices from *Glud1*^*lox/lox*^ (filled bars) and CNS-*Glud1^–/–^* (open bars) mice following metabolism of [U-^13^C]glucose *via* subsequent turns in the TCA cycle. Percent of alanine and lactate being M + 3 labeled **(A)** and the percent of citrate M + 5, citrate M + 6, glutamate M + 4, glutamate M + 5, glutamine M + 4, glutamine M + 5, GABA M + 4, and aspartate M + 4 **(B)**. Hippocampal slices from *Glud1*^*lox/lox*^ and CNS-*Glud1^–/–^* mice were incubated in the presence of [U-^13^C]glucose for 60 min as detailed in Materials and methods. The enrichment was determined from GC-MS analysis of slice extracts as detailed in Materials and methods. Results are averages ± SEM (*n* = 10), and the asterisk indicates a statistically significant difference between *Glud1*^*lox/lox*^ and CNS-*Glud1^–/–^* mice (*P* < 0.05). Cit, Citrate; Glu, Glutamate; Gln, Glutamine; GABA, γ-aminobutyric acid; Asp, Aspartate.

Glucose metabolism was assessed in slices from the CNS-*Glud1^–/–^* and *Glud1*^*lox/lox*^ mice employing [U-^13^C]glucose. The labeling into [U-^13^C]alanine, i.e., alanine M + 3, was markedly increased upon deletion of GDH while the ^13^C labeling in M + 3 lactate was similar in hippocampal slices from the two genotypes (Figure 1A). In addition, the ^13^C labeling in glutamate was slightly but significantly decreased in the first turn of tricarboxylic acid TCA cycle metabolism in brain slices lacking GDH (results not shown) and this effect was enhanced upon successive turns of TCA cycle metabolism (Figure 1B). As expected, this demonstrates an importance of GDH for glutamate synthesis. The ^13^C labeling in all other metabolites was unaffected by the absence of GDH (Figure 1B). The data obtained in cortex slices showed similar changes indicating that cortex and hippocampus were equally affected by the absence of GDH (results not shown).

### ^15^N Labeling From ^15^NH_4_Cl in Cortex Slices From CNS-*Glud1^–/–^* Mice

**FIGURE 2:**
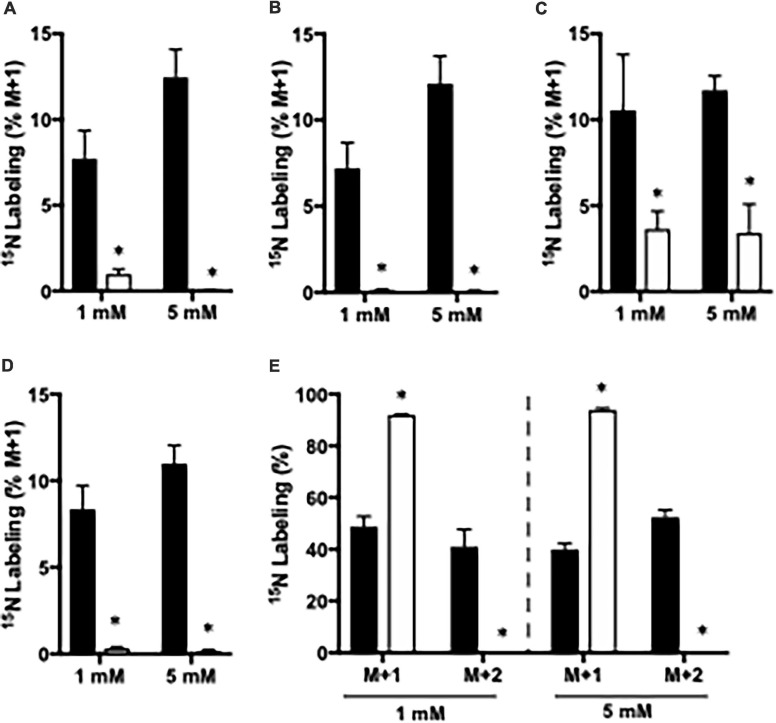
The percent ^15^N labeling in metabolites in cortical slices from *Glud1*^*lox/lox*^ (filled bars) and CNS-*Glud1^–/–^* mice (open bars) mice following incubation in the presence of 1 or 5 mM ^15^NH_4_Cl for 60 min as detailed in Materials and methods. The percent mono-labeled glutamate **(A)**, aspartate **(B)**, alanine **(C)**, GABA **(D)** as well as the percent of glutamine being mono- or double labeled **(E)** was determined from GC-MS analysis of slice extracts as detailed in Materials and methods. Results are averages ± SEM (*n* = 5–8), and the asterisk indicates a statistically significant difference between *Glud1*^*lox/lox*^ and CNS-*Glud1^–/–^* mice (*P* < 0.05).

The importance of GDH and GS for ammonia fixation was assessed by incubating cortical slices from CNS-*Glud1^–/–^* and *Glud1*^*lox/lox*^ mice. As mentioned above, ^15^NH_4_^+^ may be fixated into glutamate by GDH giving rise to glutamate M + 1, and this labeling is a prerequisite for generation of M + 1 labeling in alanine, aspartate, and GABA, and M + 2 labeling in glutamine. In slices from CNS-*Glud1^–/–^* mice the M + 1 labeling is abolished in glutamate, aspartate, and GABA and so is the M + 2 labeling in glutamine (Figures 2A–E). This clearly demonstrates that ammonia fixation *via* GDH is abolished in the hippocampal slices from CNS-*Glud1^–/–^* mice verifying the knockout of the gene. In alanine, the labeling depends on fixation of ammonia into glutamate prior to transamination into alanine, and as labeling is extinguished in glutamate in slices from CNS-*Glud1^–/–^* mice, the 3% labeling observed in alanine is likely an artifact (Figure 2C). The approximately 90% labeling in M + 1 glutamine in cortex slices from CNS-*Glud1^–/–^* mice clearly demonstrates a significant role of GS for ammonia fixation. In cortex slices from *Glud1*^*lox/lox*^ there is a concentration dependent increase in the incorporation of ^15^NH_4_^+^ into glutamate, aspartate, alanine, and GABA increasing from app. 8% when incubated with 1 mM ^15^NH_4_Cl to app. 12% when incubated with 5 mM ^15^NH_4_Cl (Figures 2A–D, respectively). Similarly, an increase was observed in M + 2 glutamine which increased from app. 45% when incubated with 1 mM ^15^NH_4_Cl to app. 55% when incubated with 5 mM ^15^NH_4_Cl while M + 1 was correspondingly decreased (Figure 2E).

### Effect of Ammonia Exposure in Brain Slices From Glud1^*lox/lox*^ Mice

**FIGURE 3:**
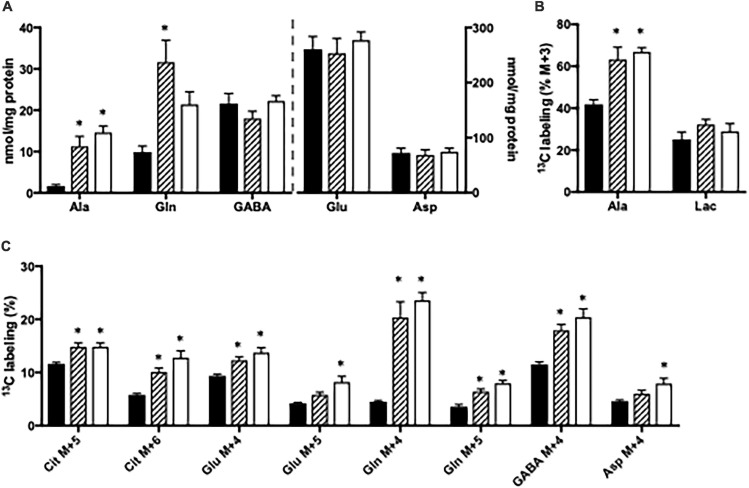
Hippocampal slices from *Glud1*^*lox/lox*^ mice were incubated in medium containing 5 mM [U-^13^C]glucose and 0 mM (control; filled bars), 1 mM (hatched bars), or 5 mM (open bars) NH_4_Cl. The effect of ammonia treatment on the amounts (nmol/mg protein) of alanine, glutamine, GABA, glutamate, and aspartate **(A)** and the percent ^13^C labeling originating from [U-^13^C]glucose in alanine M + 3 and lactate M + 3 **(B)** and in citrate M + 5, citrate M + 6, glutamate M + 4, glutamate M + 5, glutamine M + 4, glutamine M + 5, GABA M + 4, and aspartate M + 4 **(C)**. The hippocampal slices from *Glud1*^*lox/lox*^ mice were incubated in the presence of [U-^13^C]glucose for 60 min as detailed in Materials and methods. The amounts of metabolites were determined employing HPLC while the percent enrichment was determined from GC-MS analysis of slice extracts as detailed in Materials and methods. Results are averages ± SEM (*n* = 6–10), and the asterisk indicates a statistically significant difference between hippocampal slices from *Glud1*^*lox/lox*^ mice incubated in the absence and presence of 1 or 5 mM NH_4_Cl (*P* < 0.05). Ala, Alanine; Lac, Lactate; Cit, Citrate; Glu, Glutamate; Gln, Glutamine; GABA, γ-aminobutyric acid; Asp, Aspartate.

The content of amino acids was determined in cortical and hippocampal slices from *Glud1*^*lox/lox*^ mice incubated in the absence or presence of ammonia. Upon ammonia exposure, the contents of alanine and glutamine were significantly increased in hippocampal slices from *Glud1*^*lox/lox*^ mice while the contents of glutamate, aspartate, and GABA were similar (Figure 3A). Glucose metabolism was assessed in slices from the *Glud1*^*lox/lox*^ mice employing [U-^13^C]glucose. The labeling into [U-^13^C]alanine, i.e., alanine M + 3, was markedly increased upon ammonia exposure while the ^13^C labeling in lactate M + 3 was not significantly altered by the presence of ammonia (Figure 3B). Glucose metabolism in the first turn of the TCA cycle was unaffected by ammonia exposure (results not shown). In addition, when incubated in the presence of ammonia an immense increase was observed in M + 4 glutamine (Figure 3C), and this increase was similar independent of the ammonia concentration. M + 4 Glutamine is produced when labeled glucose enters the TCA cycle *via* both PC and PDH to generate M + 5 citrate and subsequently conversion *via* TCA cycle metabolism and the concerted actions of GDH and GS. Labeling in other metabolites generated *via* this pathway and/or successive cycling in the TCA cycle were concentration dependently increased upon ammonia exposure (Figure 3C). The data obtained in slices from cortex showed similar changes indicating that cortex and hippocampus were equally affected (results not shown).

### Effect of Ammonia Exposure in Brain Slices From CNS-*Glud1^–/–^* Mice

**FIGURE 4:**
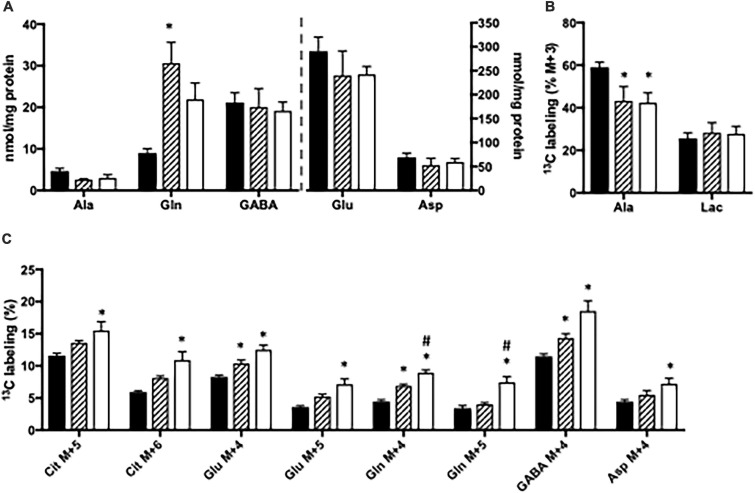
Hippocampal slices from CNS-*Glud1^–/–^* mice were incubated in medium containing 5 mM [U-^13^C]glucose and 0 mM (control; filled bars), 1 mM (hatched bars), or 5 mM (open bars) NH_4_Cl. The effect of ammonia treatment on the amounts (nmol/mg protein) of alanine, glutamine, GABA, glutamate, and aspartate **(A)** and the percent ^13^C labeling originating from [U-^13^C]glucose in alanine M + 3 and lactate M + 3 **(B)** and in citrate M + 5, citrate M + 6, glutamate M + 4, glutamate M + 5, glutamine M + 4, glutamine M + 5, GABA M + 4, and aspartate M + 4 **(C)**. The hippocampal slices from CNS-*Glud1^–/–^* mice were incubated in the presence of [U-^13^C]glucose for 60 min as detailed in Materials and methods. The amounts of metabolites were determined employing HPLC while the percent enrichment was determined from GC-MS analysis of slice extracts as detailed in Materials and methods. Results are averages ± SEM (*n* = 6–10), and the asterisk indicates a statistically significant difference from hippocampal slices from CNS-*Glud1^–/–^* mice incubated in the absence and presence of 1 or 5 mM NH_4_Cl while a number sign indicates a statistically significant difference between hippocampal slices from CNS-*Glud1^–/–^* mice incubated in 1 and 5 mM NH_4_Cl (*P* < 0.05). Ala, Alanine; Lac, Lactate; Cit, Citrate; Glu, Glutamate; Gln, Glutamine; GABA, γ-aminobutyric acid; Asp, Aspartate.

The content of amino acids was determined in cortical and hippocampal slices from CNS-*Glud1^–/–^* mice incubated in the absence or presence of ammonia. Upon ammonia exposure, the content of glutamine was significantly increased in hippocampal slices from CNS-*Glud1^–/–^* mice (Figure 4A). Interestingly, the alanine content in hippocampal slices from CNS-*Glud1^–/–^* mice (Figure 4A) did not increase as observed in slices from the *Glud1*^*lox/lox*^ mice, but instead tended to decrease. No significant changes were seen in the contents of glutamate, aspartate, and GABA (Figure 4A). In contrast to the findings observed using brain slices from the *Glud1*^*lox/lox*^ mice, the labeling into M + 3 alanine from [U-^13^C]glucose was reduced upon ammonia exposure of hippocampal slices from CNS-*Glud1^–/–^* mice (Figure 4B). No significant difference was observed in lactate M + 3 when hippocampal slices from the CNS-*Glud1^–/–^* mice were exposed to ammonia. Glucose metabolism in the first turn of the TCA cycle was unaffected by ammonia exposure (results not shown). Labeling in other metabolites generated *via* pyruvate carboxylation and/or successive cycling in the TCA cycle was concentration dependently increased upon ammonia exposure (Figure 4C). Interestingly, this included glutamine M + 4, and the immense increase observed in M + 4 glutamine in slices from the *Glud1*^*lox/lox*^ mice in the presence of ammonia (Figure 3C) was not present in slices from CNS-*Glud1^–/–^* mice. The data obtained in cortex slices showed similar changes indicating that cortex and hippocampus were equally affected by the concentration of ammonia (results not shown).

## Discussion

Dehydrogenase (GDH) constitutes a pivotal regulatory point that links energy- and amino acid metabolism including the neuroactive amino acids. Compared to aminotransferases, GDH is unique since it catalyzes a reaction that can lead to a net supply of TCA cycle intermediates, i.e., anaplerosis and, at the same time, is an essential player in ammonia homeostasis. Here, we employed acutely isolated cortical and hippocampal slices from CNS-*Glud1^–/–^* and littermate *Glud1*^*lox/lox*^ control mice to assess the role of GDH for ammonia fixation and amino acid homeostasis during hyperammonemia.

### GDH Is Important for Glutamate Metabolism

Ablation of GDH expression in brain slices is compensated for mainly by ALAT that converts glutamate and pyruvate into α-ketoglutarate and alanine, thereby providing a pathway for glutamate to enter the TCA cycle. This is observed from the higher content of alanine and the higher percent labeling in alanine M + 3 in acutely isolated brain slices from CNS-*Glud1^–/–^* compared to in *Glud1*^*lox/lox*^ control mice (Table 1 and Figure 1). In addition, the generation of glutamate from [U-^13^C]glucose *via* glycolysis and subsequent turns in the TCA cycle is hampered by the absence of GDH as observed by a lower labeling in glutamate M + 4 and M + 5 in acutely isolated brain slices from CNS-*Glud1^–/–^* compared to in *Glud1*^*lox/lox*^ control mice. All other metabolites generated from metabolism of [U-^13^C]glucose *via* glycolysis and both the first and subsequent turns of the TCA cycle were similar in acutely isolated brain slices from CNS-*Glud1^–/–^* compared to in *Glud1*^*lox/lox*^ control mice as were the amounts of glutamate, glutamine, aspartate and GABA. Although some studies reported that the Nestin-Cre transgene may induce a metabolic phenotype related to lower insulin sensitivity and reduced growth hormone production leading to a reduction in body weight gain (Briancon et al., 2010; Harno et al., 2013) such effect was not observed by others (Bence et al., 2006). Discrepancies between laboratories regarding body growth suggest that Nestin-Cre transgene may render animals more susceptible to factors such as the genetic background, supporting the non-consistent nature of this phenotype. However, we have previously monitored and reported body weights of our mouse lines CNS-*Glud1^–/–^* and littermate *Glud1*^*lox/lox*^ control mice, i.e., Nestin-Cre heterozygous carriers versus Nestin-Cre free mice on the same mixed genetic background (C57BL/6J x 129/Sv). The results demonstrate that starting at 9 weeks of age onward, body weights are similar for both genotypes (Karaca and Maechler, 2014). In the present study, we used CNS-*Glud1^–/–^* and littermate *Glud1*^*lox/lox*^ control mice in order to homogenize a mixed genetic background and additionally mice were aged 9–20 weeks; thereby avoiding the body weight difference issue. Moreover, while pathways related to glutamate metabolism were altered in brain slices from CNS-*Glud1^–/–^* compared to in those from littermate *Glud1*^*lox/lox*^ control mice as expected, other aspects of glucose metabolism were unaltered. This clearly demonstrates that possible alterations in insulin sensitivity mediated by the Nestin-Cre driver were insignificant in this setting.

### GDH Is Important for *de novo* Synthesis of Glutamine

It has repeatedly been demonstrated that GS plays a prominent role for fixation of ammonia and elevated glutamine levels in the brain and blood are key features of the pathogenesis of hyperammonemia and hepatic encephalopathy (Tofteng et al., 2006). In order to sustain continuous ammonia fixation and glutamine synthesis, *de novo* synthesis of glutamate from glucose is required to provide a substrate for glutamine synthesis by GS. This process involves entry of pyruvate into the TCA cycle *via* both PC and PDH, metabolism *via* the TCA cycle and the subsequent conversion to glutamate by GDH or an aminotransferase. Increased PC activity has been demonstrated in co-cultures of neurons and astrocytes upon ammonia exposure and *in vivo* in rats during acute liver failure (Zwingmann et al., 2003; Dadsetan et al., 2011). However, although GDH generally plays a key role in *de novo* synthesis of glutamate and glutamine, the importance of GDH in this function during hyperammonemia has acquired little attention.

**FIGURE 5:**
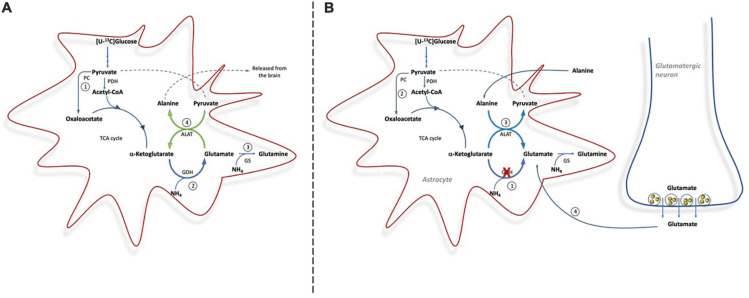
Cartoon demonstrating the metabolic pathways involved in ammonia detoxification in brain slices from *Glud1*^*lox/lox*^ mice **(A)** and from CNS-*Glud1^–/–^* mice **(B)**. **(A)**: In brain slices from *Glud1*^*lox/lox*^ mice glucose enters the TCA cycle *vi*a PDH and PC for *de novo* synthesis of glutamate (1), GDH is important for ammonia fixation and *de novo* synthesis of glutamate (2), glutamate is used as precursor for glutamine synthesis in astrocytes (3), and The concerted action of GDH and ALAT provides a mechanism for continuous fixation and disposal of ammonia as alanine is released from the brain (4). **(B)**: In brain slices from CNS-*Glud1^–/–^* mice, the lack of GDH impairs the possibility for *de novo* synthesis of glutamate (1), which results in less glucose entering the TCA *via* PC (2), and the direction of ALAT is reversed in order to sustain glutamate synthesis that can serve as precursor for glutamine synthesis (3). In addition, neurotransmitter glutamate cleared from the synapse by astrocytes is used for glutamine synthesis (4).

Here we clearly demonstrate the importance of GDH for *de novo* synthesis of glutamine involving both PC and PDH upon ammonia exposure (see Figure 5). This is observed from the immense increase in the percent of glutamine being M + 4 labeled from [U-^13^C]glucose upon ammonia exposure in slices from *Glud1*^*lox/lox*^ mice; an increase which was less pronounced in slices from CNS-*Glud1^–/–^* mice. The increase in glutamine M + 4 in brain slices from *Glud1*^*lox/lox*^ mice is dependent on a significant amount of glucose being utilized for *de novo* glutamate synthesis in astrocytes compatible with the increase in glucose uptake previously demonstrated when cortical slices from cats were exposed to elevated ammonia concentrations (McKhann and Tower, 1961). That glucose is used for *de novo* glutamate synthesis during hyperammonemia is further supported by a previous study demonstrating that PC activity induced by hyperammonemia is abolished if GS is inhibited (Dadsetan et al., 2013). As glucose is used for glutamine synthesis and does not involve complete metabolism in the TCA cycle, the requirement for oxygen consumption in the electron transport chain is not increased despite the higher glucose uptake. This may explain the mismatch in glucose and oxygen consumption observed in cat brain slices upon ammonia exposure where accelerated glucose uptake was accompanied by a decrease in oxygen consumption (McKhann and Tower, 1961). It should be noted though, that a PET study employing [^11^C]acetate demonstrated that oxidative metabolism in astrocytes was similar in patients with liver cirrhosis and in healthy controls (Iversen et al., 2014). This may rely on acetate which, in contrast to glucose, is unable to sustain *de novo* synthesis of TCA cycle intermediates as it is not a substrate for PC, and hence its metabolism is likely not increased upon hyperammonemia. Based on the percent ^13^C labeling observed in glutamate M + 4 and aspartate M + 4, that are concentration dependently increased by ammonia exposure, our data suggest that neuronal TCA cycle metabolism is stimulated rather than reduced by ammonia, a finding compatible with previous reports (Johansen et al., 2007; Leke et al., 2011).

*De novo* synthesis of glutamine involving GDH activity in astrocytes constitutes an important ammonia fixating pathway in brain slices with preserved GDH activity (i.e., from *Glud1*^*lox/lox*^ control mice). In contrast, astrocytes in brain slices from CNS-*Glud1^–/–^* mice lacking GDH are unable to perform *de novo* synthesis of glutamate which can serve as precursor for glutamine synthesis. This is observed by the much lower percent ^13^C labeling in glutamine M + 4 in slices from CNS-*Glud1^–/–^* mice compared to slices from *Glud1*^*lox/lox*^ control mice. Actually, in slices from CNS-*Glud1^–/–^* mice the ^13^C labeling in glutamine M + 4 displays a similar pattern as that in glutamate M + 4 and aspartate M + 4 generated when [U-^13^C]glucose is metabolized *via* successive turns in the TCA cycle. This implies that the glutamate pool serving as precursor for glutamine synthesis in slices from CNS-*Glud1^–/–^* mice originates from the neuronal compartment when astrocytes are incapable of performing *de novo* synthesis of glutamate due to lack of GDH expression (Figure 5B). It is expected that the glutamate pool serving as glutamine precursor is the neurotransmitter pool of glutamate which following vesicular release and interaction with receptors in the postsynaptic membrane is cleared from the extracellular space mainly by transporters located in the astrocytic membrane (Danbolt, 2001; Waagepetersen et al., 2005; Furness et al., 2008). The fact that neuronal glutamate sustains glutamine synthesis during hyperammonemia in slices from CNS-*Glud1^–/–^* mice likely result in a depletion of neurotransmitter glutamate upon sustained ammonia exposure as inferred by the trend toward a decrease in glutamate content. Hence, our results imply that depletion of glutamate content may be accelerated by the lack of GDH. Interestingly, a decrease in cortical glutamate content during hyperammonemia and acute liver failure was previously reported in a study performed in rats (Danbolt, 2001). Based on this it was proposed that ammonia induced depletion of neurotransmitter glutamate may contribute to the pathogenesis of hepatic encephalopathy (Bosman et al., 1990) as proper brain function is highly dependent on a tight balance in the activity of excitatory and inhibitory neurotransmitters (Sonnewald and Kondziella, 2003). An attenuation of glutamatergic activity during hepatic encephalopathy is compatible with the enhanced GABA tone which was observed during hepatic encephalopathy (Schafer and Jones, 1982; Jones and Basile, 1998). Here we also see that the ^13^C labeling in GABA is much higher than in glutamate in both genotypes, likely reflecting that not only neuronal glutamate but also glutamine originally labeled in the astrocytic compartment serve as important precursors for GABA synthesis as previously demonstrated (Bradford et al., 1978; Battaglioli and Martin, 1991; Sonnewald et al., 1993; Waagepetersen et al., 2001; Walls et al., 2011).

### GDH Is Important for Fixation of Ammonia

GS has repeatedly been reported to be the quantitatively most important enzyme for ammonia fixation in the brain (Cooper et al., 1979; Zwingmann et al., 2003; Dadsetan et al., 2013; Thrane et al., 2013). Our findings support a prominent role of GS for ammonia fixation as the glutamine pool in cortical slices from both CNS-*Glud1^–/–^* and *Glud1*^*lox/lox*^ mice was approximately 90% ^15^N labeled when incubated in the presence of ^15^NH_4_Cl. However, we also demonstrate a significant role of GDH for fixation of ^15^NH_4_^+^ as evident from glutamine being approximately 50% double labeled in slices from *Glud1*^*lox/lox*^ mice. The importance of GDH for ammonia fixation is further underlined by a significant labeling in the glutamate pool of approximately 10% in slices from *Glud1*^*lox/lox*^ mice which was completely eliminated in slices from CNS-*Glud1^–/–^* mice. It should be noted that ammonia fixation *via* GDH appears to be more prominent in astrocytes as double labeling of glutamine is more pronounced than mono-labeling of its precursor glutamate, indicating that a small pool of glutamate with a high turnover contributes to glutamine synthesis in astrocytes as previously proposed (Berl et al., 1962; Cooper et al., 1979). However, ammonia fixation by GDH appears to be important in neurons as well since a significant mono-labeling from ^15^NH_4_Cl was detected in metabolites representing the neuronal compartment, i.e., glutamate, aspartate and GABA (Ottersen and Storm-Mathisen, 1985; Kaufman et al., 1991; Ottersen et al., 1992). Interestingly, the extent of ^15^N labeling of glutamate and the extent of double labeling in glutamine were both augmented with increasing ammonia concentrations. This confirms that ammonia concentration dependently increases both GDH and GS activity as previously observed in a study employing cell cultures incubated in the presence of ^15^NH_4_Cl (Dadsetan et al., 2013). That GDH becomes increasingly important for ammonia fixation with higher ammonia concentrations may also reflect that the change in the direction of GDH from oxidative deamination of glutamate to reductive amination of α-ketoglutarate is determined by the ammonia concentration, compatible with the K_*m*_ of GDH for ammonia being in the range of 15 mM (Zaganas et al., 2001). Alternatively, an increased importance of GDH with higher ammonia concentrations may result from the capacity of GS being limiting during hyperammonemia as previously suggested (Cooper et al., 1979, 1985; Bosman et al., 1990; Kanamori et al., 1996). In support of this, an augmented GDH activity was previously demonstrated to compensate at least to some extent for the lack of ammonia detoxification by glutamine production when GS was inhibited by MSO in rats (Dadsetan et al., 2013).

The concerted action of GDH and ALAT has repeatedly been demonstrated to constitute an important ammonia scavenging pathway *in vitro* and *in vivo* and flux through this pathway is intensified when GS is inhibited (Leke et al., 2011; Dadsetan et al., 2013). The coupling of GDH and ALAT activity is advantageous since GDH conveys ammonia fixation (see Figure 5). The subsequent action of ALAT transfers ammonia to alanine which can be released from the brain (Hawkins et al., 2006). Concomitantly, ALAT converts glutamate to α-ketoglutarate thereby ensuring α-ketoglutarate availability and providing a means for continuous ammonia fixation by GDH. In order to continue this process, a tight coupling of GDH and ALAT activity is necessary and accordingly it has been suggested that GDH and ALAT form transient complexes as demonstrated by Fahien et al. (1977). Also, in favor of a concerted action of GDH and ALAT, the cerebral content of alanine was elevated in an animal model of fulminant liver failure (Swain et al., 1992) and in patients with fulminant hepatic failure (Tofteng et al., 2006). Here, we confirm the importance of GDH acting in concert with ALAT to constitute an ammonia scavenging pathway, since both alanine content and the percent alanine being M + 3 labeled from [U-^13^C]glucose in slices from *Glud1*^*lox/lox*^ mice were markedly increased upon ammonia exposure (Figure 5A). Such increase was eliminated in slices from CNS-*Glud1^–/–^* mice clearly demonstrating the importance of GDH for ammonia fixation. In fact, upon ammonia exposure in brain slices lacking GDH activity, ALAT seems to operate in the opposite direction, i.e., consuming alanine (Figure 5B). This is observed from the decrease in percent ^13^C labeling in alanine M + 3 from [U-^13^C]glucose combined with a trend toward a lower alanine content in slices from CNS-*Glud1^–/–^* mice. Reversal of ALAT gives rise to glutamate production which likely alleviates ammonia fixation by serving as precursor for glutamine synthesis. This further supports the importance of GDH for ammonia fixation as reversal of ALAT serves as a compensatory mechanism in slices lacking GDH.

It should be noted that ALAT activity not only compensates for lack of GDH during hyperammonemia but also when slices from CNS-*Glud1^–/–^* mice were incubated in the absence of ammonia. However, in the absence of ammonia ALAT operates in the direction toward alanine production, as evident from the elevated alanine content and percent alanine M + 3 from [U-^13^C]glucose measured in slices from CNS-*Glud1^–/–^* mice compared to in those from *Glud1*^*lox/lox*^ mice. This action of ALAT concomitantly converts glutamate to α-ketoglutarate and thereby resembles the physiological direction of GDH in the brain (Kaufman et al., 1991) thereby providing the opportunity for metabolizing glutamate in the TCA cycle for energy production. This is in line with a previous study demonstrating the importance of GDH for sustaining neuronal oxidative energy metabolism (Hohnholt et al., 2018). In addition, the fact that results obtained from cortex slices were similar to those obtained from hippocampal slices (results not shown) demonstrates that the two brain areas do not differ with regard to the metabolic pathways being involved in ammonia detoxification.

We demonstrate a prominent role of GDH for ammonia fixation and its coupling to ALAT activity offers an enduring ammonia scavenging pathway (Figure 5). It might be expected that AAT would be able to play a similar role as ALAT, and evidence for activity of this pathway was obtained from a significant incorporation of ^15^N into aspartate reflecting the extent of labeling in its precursor glutamate. However, the ^13^C labeling in aspartate M + 4 reflected the increase in TCA cycle intermediates, and in contrast to the alanine content, aspartate content was not increased upon ammonia exposure. The differences in ALAT and AAT activity coupled to GDH activity may rely on 1) aspartate being neuroactive and high affinity transporters ensure that aspartate is kept inside the cell whereas alanine can be released from the brain *via* a transport system in the blood-brain barrier (Hawkins et al., 2006) and hence alanine can serve as an ammonia sink, and 2) AAT activity does not lead to a net increase in the pool of TCA cycle intermediates while the action of ALAT does.

### Clinical Perspectives

The role of GDH in hyperammonemia, and the concerted action with ALAT provides novel knowledge that may be used as basis for treatment of conditions involving hyperammonemia. Besides hepatic encephalopathy, elevated ammonia levels in blood were demonstrated in patients with non-alcoholic fatty liver disease (NAFLD) even at early stages (De Chiara et al., 2018). NAFLD is a lifestyle induced spectrum of liver disease ranging from steatosis, through non-alcoholic steatohepatitis (NASH) to cirrhosis, and is characterized by fatty acid uptake, *de novo* fatty acid synthesis and reduced β-oxidation leading to increased liver fat in patients that are not consuming excessive alcohol (De Chiara et al., 2018). The prevalence of NAFLD is high and a future increase is expected based on the fact that the presence of coexisting risk factors such as diabetes, metabolic syndrome, and obesity increases the risk of NAFLD (Piazzolla and Mangia, 2020). Hence, the findings of the present study may become valuable with regard to understanding the pathogenesis and development of treatments for several different hepatic diseases.

## Conclusion

Altogether we have demonstrated a prominent role of GDH for ammonia fixation during hyperammonemia. Most importantly, GDH serves as a means to uphold *de novo* glutamate synthesis from glucose and thereby provide a substrate for glutamine production and this process concomitantly contributes to ammonia fixation. In addition, we demonstrated that GDH is coupled to ALAT which does not only operate in a cyclic manner to fixate ammonia and subsequently regenerate α-ketoglutarate from glutamate, but simultaneously produce alanine which is capable of leaving the brain. Hence, the successive operation of GDH and ALAT constitutes a pathway leading to elimination of ammonia from the brain in the form of alanine.

## Data Availability Statement

The original contributions presented in the study are included in the article/supplementary material, further inquiries can be directed to the corresponding author.

## Ethics Statement

The animal study was reviewed and approved by The Animal Experiments Inspectorate Ministry of Environment and Food of Danish Veterinary and Food Administration (only breeding of transgenic mice needed approval). Written informed consent was obtained from the owners for the participation of their animals in this study.

## Author Contributions

AW, CV, and HW: study concept and design. LA and CV: acquisition of data. AW, JN, LA, CV, HW, AS, HV, and PO: analysis and interpretation of data. AW, JN, LA, HW, AS, HV, and PO: drafting of the manuscript. CV, LA, JN, HW, AS, PM, HV, PO, and AW: critical revision of the manuscript for important intellectual content. AW, LA, JN, and CV: statistical analysis. AW: study supervision. AW and HV: funding acquisition. PM (mice): administrative, technical, or material support. All authors contributed to the article and approved the submitted version.

## Conflict of Interest

The authors declare that the research was conducted in the absence of any commercial or financial relationships that could be construed as a potential conflict of interest.
